# Evolutionary algorithm based heuristic scheme for nonlinear heat transfer equations

**DOI:** 10.1371/journal.pone.0191103

**Published:** 2018-01-19

**Authors:** Azmat Ullah, Suheel Abdullah Malik, Khurram Saleem Alimgeer

**Affiliations:** 1 Junior Engineer (Instrumentation), Oil & Gas Development Company Limited (OGDCL), Islamabad, Pakistan; 2 Department of Electrical Engineering, Faculty of Engineering and Technology, International Islamic University, Islamabad, Pakistan; 3 Department of Electrical Engineering, COMSATS Institute of Information Technology, Islamabad, Pakistan; China University of Mining and Technology, CHINA

## Abstract

In this paper, a hybrid heuristic scheme based on two different basis functions i.e. Log Sigmoid and Bernstein Polynomial with unknown parameters is used for solving the nonlinear heat transfer equations efficiently. The proposed technique transforms the given nonlinear ordinary differential equation into an equivalent global error minimization problem. Trial solution for the given nonlinear differential equation is formulated using a fitness function with unknown parameters. The proposed hybrid scheme of Genetic Algorithm (GA) with Interior Point Algorithm (IPA) is opted to solve the minimization problem and to achieve the optimal values of unknown parameters. The effectiveness of the proposed scheme is validated by solving nonlinear heat transfer equations. The results obtained by the proposed scheme are compared and found in sharp agreement with both the exact solution and solution obtained by Haar Wavelet-Quasilinearization technique which witnesses the effectiveness and viability of the suggested scheme. Moreover, the statistical analysis is also conducted for investigating the stability and reliability of the presented scheme.

## Introduction

Most of the real world problems especially heat transfer equations which emerge in many scientific and engineering fields possess nonlinear behavior, are modeled by nonlinear ordinary differential equations and hence attained an ever increasing attention of scientists and engineers towards the solution of these nonlinear problems. Consequently several analytical and numerical techniques have been suggested by researchers for solving nonlinear heat transfer equations as quoted under references [1–10].

The heat transfer equations have major practical importance in cooling of electronic components, cooling of heated stirred vessels and heated parts of the space vehicles etc [4].

This work is mainly concerned to present a heuristic scheme which is stochastic in nature for the study of two nonlinear heat transfer equations. First equation refers to a well known boundary value temperature distribution equation in a lumped system of combined convection-radiation that can be described by the following mathematical model:

$$x^{\prime \prime }-\delta x^{4}=0$$ (1)

With the boundary conditions:

x′(0)=aandx(1)=b

While second equation is regarded as an initial value cooling equation of a lumped system by combined convection and radiation whose governing equation is given by:

$$x^{\prime }+x+\delta x^{4}=0$$ (2)

With the initial condition:

x(0)=c

where *a*, *b*, *c* and *δ* are real valued constants.

Researchers put forward an extensive collection of techniques for obtaining the numerical solution of such nonlinear heat transfer equations. To highlight a few, Ganji et al. [1] tailored nonlinear heat transfer equations by using homotopy perturbation method (HPM) and variational iteration method (VIM). A. Atangana in [2] adopted a new iterative analytical technique along with the stability, convergence and the uniqueness analysis of adopted technique for dealing with nonlinear fractional partial differential equations arising in biological population dynamics system. Yang Juan-Cheng et al. in [3] employed Direct Numerical Simulation (DNS) to look at the mechanism of heat transfer enhancement (HTE) with the findings that DNS procedure is trustworthy. Abbasbandy [4] encountered nonlinear heat transfer equations by using homotopy analysis method (HAM). Kolade M. Owolabi et al. in [5] estimated the fractional Schrodinger equation with the Riesz fractional derivative by making use of the improved exponential time differencing Runge-Kutta scheme with fourth-order accuracy and realized a different distribution of the complex wave functions both for the focusing and defocusing cases. R. A. Khan in [6] adopted generalized approximation method (GAM) and found that GAM perform quite effectively irrespective of dependency on small parameter as in case of HPM and PM. Dehghan et al. [7] employed semi-analytical methods i.e. homotopy perturbation method (HPM) and finite difference method (FDM) which enhances heat transfer and validates semi-analytical methods for study of heat transfer rates while Saeed et al. [8] obtained the solutions of nonlinear heat transfer equations by using Haar Wavelet-Quasilinearization Technique.

Because of limitations in accuracy and efficiency of these classical analytical and numerical methods, the evolutionary computing techniques (ECT) have demonstrated their importance, investigated thoroughly and applied successfully to solve nonlinear problems in engineering and applied science domain [11–12]. For instance, Arqub et al. [13, 14] considered continuous GA based technique for dealing with boundary value problem, Troesch’s and Bratu’s Problems. Malik et al. [15, 16] taken into account an evolutionary computing scheme of hybrid genetic algorithm (HGA) for solving biochemical reaction and Singular Boundary Value Problems Arising in Physiology. Kadri et al. [17] solved nonlinear heat conduction problems by using genetic algorithm (GA). Khan et al. [18, 19] solved fractional order system of Bagley-Torvik equation and differential equations of first orders by using HGA and swarm intelligence approaches with the findings that the proposed evolutionary computing techniques are reliable and effective. Arqub et al. in [20] investigated the efficiency, accuracy and convergence analysis of the continuous genetic algorithm by solving a class of nonlinear systems of second-order boundary value problems.

In this paper, nonlinear heat convection-radiation equations have been treated numerically by using a heuristic scheme which is stochastic in nature and hybridized with Log Sigmoid and Bernstein Polynomial basis functions with unknown coefficients. The equation representing nonlinear heat transfer phenomena is converted into an error minimization problem. The hybrid approach of genetic algorithm is exploited for solving the error minimization problem and to obtain the unknown coefficients that further gives the numerical solution of the problem under consideration.

## Methodology overview

In this section, the research methodology for the hybridization of Evolutionary Algorithm (EA) with two different basis functions i.e. Log Sigmoid and Bernstein polynomials for treating nonlinear heat transfer equations is presented in detail as under.

### Methodology overview for EA hybridization with Log Sigmoid Basis Function

For the hybridization of EA with Log Sigmoid Basis Function, we assume that the approximate numerical solution *x*(*t*) of Eq (1) and its first and second derivatives that is *x*′(*t*) and *x*″(*t*), is linear Combination of some log sigmoid basis functions that can be represented by the following equations:-

$$x(t)=\sum_{i=1}^{k}\alpha_{i}\phi \left(\omega_{i}t+\beta_{i}\right)$$ (3)

$$x^{\prime }\left(t\right)=\sum_{i=1}^{k}\alpha_{i}\omega_{i}\phi^{\prime }\left(\omega_{i}t+\beta_{i}\right)$$ (4)

$$x^{\prime \prime }\left(t\right)=\sum_{i=1}^{k}\alpha_{i}\omega_{i}{}^{2}\phi^{\prime \prime }\left(\omega_{i}t+\beta_{i}\right)$$ (5)

Where *ϕ*(*t*) is log sigmoid function defined by

$$\phi (t)=\frac{1}{1+e^{-t}}$$

(*α*_*i*_, *ω*_*i*_, *β*_*i*_) are real valued unknown parameters and *k* is the number of basis functions. The given nonlinear ODE of Eq (1) is converted into an error minimization problem that provides the required unknown parameters (*α*_*i*_, *ω*_*i*_, *β*_*i*_) as follows:

$$\varepsilon_{1}=\frac{1}{N+1}\sum_{i=0}^{N}{\left(x^{\prime \prime }\left(t_{i}\right)-\delta x^{4}\left(t_{i}\right)\right)}^{2}$$ (7)

$$\varepsilon_{2}=\frac{1}{2}\left({\left(x^{\prime }\left(0\right)-a\right)}^{2}+{\left(x\left(1\right)-b\right)}^{2}\right)$$ (8)

$$\varepsilon_{j}=\varepsilon_{1}+\varepsilon_{2}$$ (9)

Where *N* is the total number of steps taken in the solution range [0, 1]. *ε*_1_ is the mean of the sum of square error of the given system (1), *ε*_2_ is the mean of the sum of square error due to boundary conditions of Eq (1) and *j* is the number of generations executed.

The minimization of Eq (9) is carried out by using Genetic Algorithm. The optimal values of unknown parameters (*α*_*i*_, *ω*_*i*_, *β*_*i*_) resulting in minimum *ε*_*j*_ are used in Eq (3) that gives the approximate numerical solution of Eq (1). The same methodology applies for obtaining the approximate numerical solution of Eq (2) using Log Sigmoid Basis Function.

### Methodology overview for EA hybridization with Bernstein Polynomial Basis Function

For the hybridization of EA with Bernstein Polynomial Basis Function, again we assume that the approximate numerical solution *x*(*t*) of Eq (1) and its first and second derivatives that is *x*′(*t*) and *x*″(*t*), is linear combination of Bernstein Polynomial (B-Polynomials) basis functions of degree *k* and can be represented by the following equations:-

$$x(t)=\sum_{i=0}^{k}\alpha_{i}B_{i,k}\left(t\right)$$ (10)

$$x^{\prime }\left(t\right)=\sum_{i=0}^{k}\alpha_{i}B_{i,k}^{\prime }\left(t\right)$$ (11)

$$x^{\prime \prime }\left(t\right)=\sum_{i=0}^{k}\alpha_{i}B_{i,k}^{\prime \prime }\left(t\right)$$ (12)

Where (*α*_0_, *α*_1_,…..*α*_*k*_) are unknown parameters and *k* is the degree of B-polynomials.

The given nonlinear ODE of Eq (1) is then converted into an error minimization problem that provides the required unknown parameters (*α*_0_, *α*_1_,…..*α*_*k*_) by using the same Eqs (7–9). The minimization of fitness function *ε*_*j*_ as defined by Eq (9) for B-Polynomials basis functions is carried out by using Genetic Algorithm. The optimal values of unknown parameters (*α*_0_, *α*_1_,…..*α*_*k*_) resulting in minimum *ε*_*j*_ achieved are then used in Eq (10) that gives the approximate numerical solution of Eq (1). The same methodology applies for obtaining the approximate numerical solution of Eq (2) using Bernstein Polynomial Basis Function.

## Heuristic optimization technique

Genetic algorithm is a widely known global heuristic search optimization technique in evolutionary computing field. Genetic algorithm is inspired by the Darwinian principle of evolution and natural selection in which stronger individuals are likely to be the winners in a competing environment.Genetic algorithm operates on a population of individuals referred to as chromosomes.Each chromosome represents a possible solution to the problem and assigned a real numbered fitness value, which is a measure of the excellence of the solution to the particular problem. Genetic algorithm starts with a randomly generated population of chromosomes, carries out a process of fitness based selection and recombination to produce the next generation. During recombination, two or more selecting parent chromosomes recombine to produce child chromosomes. This process is repeated iteratively and recursively by means of genetic operators; selection, crossover and mutation with a hope that the average fitness of the chromosomes increase until some defined stopping criteria is fulfilled. In this way, Genetic algorithm searches for the best optimum possible solution to the given problem [11–12].

Interior-Point Algorithm (IPA) is a local search optimizer which is used extensively in variety of optimization problems. IPA solves Karush-Kuhn-Tucker (KKT) equations in the system by applying either newton step or conjugate gradient (CG) step iteratively to optimize problem’s defined merit function [11, 16].

Hybrid approach of evolutionary algorithms is efficient in the sense that they are accurate and fast convergent. In hybrid approach, the optimum chromosomes found by global optimizer (GA) are given to the local optimizer (IPA) as starting point which improves the solution provided by global optimizer (GA) [11, 16].

The steps for the hybridized scheme of GA with IPA used in this work are outlined below in Table 1 [16].

**Table 1:** Steps for hybridization of GA with IPA.

**Step 1 (Population Initialization)**	Random population of N individuals or chromosomes having M genes per chromosome is generated in a bounded limit.
**Step 2 (Evaluation & Ranking)**	Evaluate each individual using problem specific fitness function and rank them proportionate to their fitness value.
**Step 3 (Stoppage Criteria)**	The algorithm keeps executing until and unless some user defined stoppage criteria is met. If the stopping criterion is satisfied then go to step 6, else repeat steps 2 to 5.
**Step4 (Selection & Reproduction)**	Based on fitness value, the chromosomes from current population are chosen as parents for new generation. These parents then produce further offspring as a result of crossover operation which became parents for next generation.
**Step 5 (Mutation)**	Mutation operation is an optional operator and execute only if no improvement in the fitness value of the generation is seen. It randomly changes the offspring resulted from crossover to find a good solution.
**Step 6 (Local Search Improvement)**	The optimum chromosomes found by GA fed to local optimizer IPA as starting point which tends to optimize the results further.

## Implementation of proposed scheme

In this section, the proposed scheme is implemented on nonlinear heat transfer equations for the validity of the suggested approach by using both Log Sigmoid and Bernstein Polynomial basis functions. MATLAB tool is used for systems simulations.

### Implementation of proposed scheme using Log Sigmoid basis function

**Problem 01**. Let us consider temperature distribution equation in combined convection-radiation lumped system: Let the system has volume *υ*, surface area Ψ, density *ρ*, specific heat *η*, *η*_*c*_ is specific heat at temperature *T*_*c*_, initial temperature *T*_0_, temperature of the convection environment *T*_*c*_ and heat transfer coefficient ℏ. The temperature distribution equation of the system is given by nonlinear boundary value problem of (1) as [8]:

$$x^{\prime \prime }-\delta x^{4}=0$$ (13)

The boundary conditions are:

$$x^{\prime }(0)=0\;\mathrm{and}\;x(1)=1$$

Where *x* = (*T*−*T*_*c*_)/(*T*_0_−*T*_*c*_) is dimensionless temperature and *δ* = *γ*(*T*−*T*_*c*_).

The approximate solution of Eq (13) is obtained in the range [0, 0.8] with increment of 0.2 and basis function *k* = 10. The results obtained are restricted within the bounds [-25, +25]. Two cases for Eq (13) have been treated by considering *δ* = 0.6 and *δ* = 2.0.

The fitness functions formulated for Eq (13) for both the cases i.e. for *δ* = 0.6 and *δ* = 2.0 are as follows:

$$\varepsilon_{j1}=\frac{1}{11}\sum_{i=1}^{11}\left(x\prime \prime \left(t_{i}\right)-0.6x^{4}{\left(t_{i}\right)}^{2}\right)+\frac{1}{2}\left({\left(x^{\prime }\left(0\right)\right)}^{2}+{\left(x\left(1\right)-1\right)}^{2}\right)$$ (14)

$$\varepsilon_{j2}=\frac{1}{11}\sum_{i=1}^{11}\left(x\prime \prime \left(t_{i}\right)-2x^{4}{\left(t_{i}\right)}^{2}\right)+\frac{1}{2}\left({\left(x^{\prime }\left(0\right)\right)}^{2}+{\left(x\left(1\right)-1\right)}^{2}\right)$$ (15)

Where *x*(*t*), *x*′(*t*), *x*″(*t*) are same as defined by Eqs (3–5) respectively.

The fitness functions in Eqs (14 and 15) are functions of unknown parameters (*α*_*i*_, *ω*_*i*_, *β*_*i*_) and these unknown parameters are achieved by employing GA, IPA and hybrid scheme of GA-IPA for minimizing Eqs (14 and 15) and accordingly the approximate solution of Eq (13) is obtained for both the values of *δ*. The parameter settings used for the execution of GA and IPA corresponding to minimum fitness *ε*_*j*1_, *ε*_*j*2_ are given in Table 2. The fitness functions have been minimized by fixing the size of chromosome i.e. total number of unknown parameters equal to 30. The values of unknown parameters obtained by GA, IPA and GA-IPA are provided in Tables 3 and 4 for case 1 and case 2 respectively. The approximate solutions achieved by GA, IPA and GA-IPA for both the cases are provided in Tables 5 and 6. The comparisons of absolute errors necessary to demonstrate the applicability of the suggested approach are provided in Tables 7 and 8.

**Table 2:** Parameter settings.

G.A		IPA	
Parameter	Setting	Parameter	Setting
Chromosome Size	30	Start Point	randn (1,30)
Population Size	[240 240]	Maximum Iterations	1000
Selection Function	Stochastic Uniform	Maximum Function Evaluations	90,000
Crossover Function	Heuristic	Derivative type	Forward Differences
Generations	1000	Hessian	BFGS
Function Tolerance	1.00E-17	Function Tolerance	1.00E-17

**Table 3:** Unknown parameters achieved by GA, IPA and GA-IPA for δ = 0.6.

Index	G.A			IPA			G.A-IPA		
(i)	α_i_	ω_i_	β_i_	α_i_	ω_i_	β_i_	α_i_	ω_i_	β_i_
**1**	-1.769632	1.257087	0.102815	0.774634	0.829575	-0.442360	-1.692160	1.168324	0.617664
**2**	3.054979	1.010789	-1.828103	2.112032	-1.539686	-2.153781	5.929072	2.291575	-6.321103
**3**	-0.639028	-0.859267	-0.037691	0.350214	2.419460	1.845233	-0.090501	0.247958	0.079951
**4**	0.895939	-0.213643	1.470376	-0.716849	-0.465514	-4.092749	1.080930	0.834442	1.762203
**5**	-0.143908	4.651567	1.460076	-2.526024	-3.040063	-7.861947	-0.509576	2.691068	3.051515
**6**	0.342426	1.960685	3.445951	-7.231108	-5.260963	-14.173051	0.183019	1.596488	3.759634
**7**	1.268470	1.170450	0.373517	-7.198005	-6.728799	-22.056574	1.183224	0.601856	-0.103722
**8**	0.190436	0.342884	0.006668	1.836145	-0.220060	-8.096166	0.994938	0.363937	-0.180168
**9**	0.073985	0.623643	-0.055706	6.576411	1.964954	-5.739731	0.799984	0.546280	-0.284968
**10**	-0.644206	1.798445	-1.013198	24.124203	0.905876	-10.581402	-2.591654	2.082568	-7.377486

**Table 4:** Unknown parameters achieved by GA, IPA and GA-IPA for δ = 2.0.

Index	G.A			IPA			G.A-IPA		
(i)	α_i_	ω_i_	β_i_	α_i_	ω_i_	β_i_	α_i_	ω_i_	β_i_
**1**	-1.164928	2.678514	-2.188226	1.748070	-1.047044	-0.116665	-1.506841	2.923555	-3.221775
**2**	0.774210	1.296735	-1.669520	1.223584	1.997789	1.816609	1.150150	1.243912	-1.951968
**3**	0.892156	-2.204351	-1.417349	1.489439	0.079010	0.491226	1.283040	-1.988222	-2.034376
**4**	-1.061263	-0.303247	-2.799944	3.510684	-1.966125	-2.449384	-1.040591	-0.223657	-2.818933
**5**	-0.333092	-0.412516	0.493098	8.262437	3.714661	-7.870645	-0.252763	-0.448212	0.489439
**6**	-0.077865	0.605382	0.600815	-0.182649	-0.082647	0.144682	-0.024551	0.612447	0.586979
**7**	-0.631578	2.141546	-0.183580	1.689271	1.348407	-1.690119	-0.746927	2.227827	-0.989887
**8**	4.009014	1.392278	-2.039139	0.353447	0.126981	-0.646432	4.564188	1.971857	-3.049711
**9**	0.310875	0.812015	0.476334	-7.941511	-0.994241	-3.087374	0.317166	0.713770	0.444734
**10**	1.002565	0.498146	-0.140206	-2.410417	0.191338	3.336692	1.108091	0.431417	-0.215144

**Table 5:** Approximate solutions by numerical methods and proposed method for δ = 0.6.

	Numerical Methods				Proposed Method x(t)		
t	x_Maple_	x_GA_	x_HPM_	x_Haar_	G.A	IPA	G.A-IPA
**0.0**	0.834542	0.963536	0.640000	0.834543	0.834049	0.834563	0.834548
**0.2**	0.840390	0.964009	0.652096	0.840391	0.839927	0.840409	0.840396
**0.4**	0.858269	0.965742	0.689536	0.858269	0.857818	0.858287	0.858275
**0.6**	0.889247	0.969893	0.755776	0.889248	0.888648	0.889265	0.889255
**0.8**	0.935346	0.979233	0.866576	0.935346	0.934877	0.935366	0.935355

**Table 6:** Approximate solutions by numerical methods and proposed method for δ = 2.0.

	Numerical Methods				Proposed Method x(t)		
t	x_Maple_	x_GA_	x_HPM_	x_Haar_	G.A	IPA	G.A-IPA
**0.0**	0.694318	0.968771	0.666667	0.694362	0.694123	0.694379	0.694527
**0.2**	0.703698	0.968804	0.625600	0.703739	0.703335	0.703761	0.703916
**0.4**	0.732894	0.969008	0.489600	0.732927	0.732322	0.732967	0.733149
**0.6**	0.785488	0.970024	0.220267	0.785510	0.784242	0.785578	0.785807
**0.8**	0.869161	0.975059	0.246400	0.869176	0.867735	0.869293	0.869632

**Table 7:** Comparison of absolute errors between numerical methods and proposed method for δ = 0.6.

	Numerical Methods [x_Exact_—x(t)]			Proposed Method		[x_Exact_—x(t)]
t	x_GA_	x_HPM_	x_Haar_	G.A	IPA	G.A-IPA
**0.0**	-1.290E-01	1.945E-01	-1.000E-06	4.927E-04	-2.093E-05	-6.216E-06
**0.2**	-1.236E-01	1.883E-01	-1.000E-06	4.627E-04	-1.931E-05	-6.341E-06
**0.4**	-1.075E-01	1.687E-01	0.000E+00	4.509E-04	-1.780E-05	-6.494E-06
**0.6**	-8.065E-02	1.335E-01	-1.000E-06	5.989E-04	-1.779E-05	-7.745E-06
**0.8**	-4.389E-02	6.877E-02	0.000E+00	4.692E-04	-1.966E-05	-9.369E-06

**Table 8:** Comparison of absolute errors between numerical methods and proposed method for δ = 2.0.

	Numerical Methods [x_Exact_—x(t)]				Proposed Method [x_Exact_—x(t)]	
t	x_GA_	x_HPM_	x_Haar_	G.A	IPA	G.A-IPA
**0.0**	-2.745E-01	2.765E-02	-4.400E-05	1.947E-04	-6.073E-05	-2.086E-04
**0.2**	-2.651E-01	7.810E-02	-4.100E-05	3.634E-04	-6.265E-05	-2.182E-04
**0.4**	-2.361E-01	2.433E-01	-3.300E-05	5.721E-04	-7.255E-05	-2.553E-04
**0.6**	-1.845E-01	5.652E-01	-2.200E-05	1.246E-03	-8.965E-05	-3.194E-04
**0.8**	-1.059E-01	6.228E-01	-1.500E-05	1.426E-03	-1.320E-04	-4.706E-04

The comparisons of absolute errors given in Tables 7 and 8 for both the cases i.e. *δ* = 0.6 and *δ* = 2.0 reveals that the proposed scheme dealt the given nonlinear problem with quite excellent accuracy than GA and HPM while the results are quite compromising with the exact results and results obtained by Haar Wavelet method which can be made more competitive by increasing the number of basis functions.

**Problem 02.** Let us consider case of cooling of a lumped system by combined convection and radiation: Let the system has volume *υ*, surface area Ψ, density *ρ*, specific heat *η*, *η*_*c*_ is specific heat at temperature *T*_*c*_, initial Temperature *T*_0_, temperature of the convection environment *T*_*c*_, emissivity *μ*, heat transfer coefficient ℏ and sink temperature is represented by *T*_*α*_ as the system loses heat through radiation. The mathematical model of the system is given by the nonlinear initial value problem of Eq (2) as [8]:

$$x^{\prime }+x+\delta x^{4}=0$$ (16)

x(0)=1

The approximate solution of Eq (16) is obtained in the range [0, 1] with a step increment of 0.1 and basis function *k* = 10. The results obtained are restricted within the bounds [-20, +20]. The fitness function is formulated as given by:

$$\varepsilon_{j}=\frac{1}{11}\sum_{i=1}^{11}\left({\left(x^{\prime }\left(t_{i}\right)+x\left(t_{i}\right)+\delta x^{4}\left(t_{i}\right)\right)}^{2}\right)+\left({\left(x\left(0\right)-1\right)}^{2}\right)$$ (17)

GA and IPA are implemented using the settings as given in Table 9. The values of unknown parameters corresponding to minimum *ε*_*j*_ for different values of δ are omitted in this case. These unknown parameters are necessary to known for obtaining the approximate solution $\hat{x}(t)$ of Eq (17). The results achieved by GA, IPA and GA-IPA are shown in Table 10. The comparison of absolute errors provided by our method is made with available methods and is given in Table 11.

**Table 9:** Parameter settings.

G.A		IPA	
Parameter	Setting	Parameter	Setting
Chromosome Size	30	Start Point	randn (1,30)
Population Size	[180 180]	Maximum Iterations	1000
Selection Function	Roulette	Maximum Function Evaluations	120,000
Crossover Function	Heuristic	Derivative type	Central Differences
Generations	1000	Hessian	BFGS
Function Tolerance	1.00E-18	Function Tolerance	1.00E-18

**Table 10:** Approximate solutions by numerical methods and proposed method for different δ and t = 0.5.

	Numerical Methods				Proposed Method x(t)		
δ	x_Exact_	x_VIM_	x_HPM_	x_Haar_	G.A	IPA	G.A-IPA
**0.0**	0.606531	0.606531	0.606531	0.606531	0.606519	0.606531	0.606528
**0.1**	0.591591	0.591617	0.591638	0.591592	0.591590	0.591591	0.591585
**0.2**	0.578023	0.578207	0.578371	0.578023	0.578006	0.578022	0.578020
**0.3**	0.565620	0.566185	0.566732	0.565620	0.565623	0.565619	0.565624
**0.4**	0.554217	0.555440	0.556720	0.554217	0.554172	0.554216	0.554213
**0.5**	0.543681	0.545868	0.548335	0.543681	0.543610	0.543679	0.543674
**0.6**	0.533903	0.537369	0.541576	0.533904	0.533818	0.533901	0.533895
**0.7**	0.524793	0.529850	0.536445	0.524793	0.524722	0.524794	0.524784
**0.8**	0.516275	0.523226	0.532940	0.516275	0.516191	0.516273	0.516267
**0.9**	0.508284	0.517412	0.531062	0.508284	0.508247	0.508282	0.508249
**1.0**	0.500765	0.512333	0.530812	0.500765	0.500626	0.500763	0.500743

**Table 11:** Comparison of absolute errors for different δ and t = 0.5.

	Numerical Methods [x_Exact_—x(t)]			Proposed Method [x_Exact_—x(t)]		
δ	x_VIM_	x_HPM_	x_Haar_	G.A	IPA	G.A-IPA
**0.0**	0.000E+00	0.000E+00	0.000E+00	1.223E-05	-1.858E-07	2.752E-06
**0.1**	-2.600E-05	-4.700E-05	-1.000E-06	1.022E-06	-5.829E-08	5.614E-06
**0.2**	-1.840E-04	-3.480E-04	0.000E+00	1.652E-05	1.322E-06	2.571E-06
**0.3**	-5.650E-04	-1.112E-03	0.000E+00	-3.373E-06	7.018E-07	-3.520E-06
**0.4**	-1.223E-03	-2.503E-03	0.000E+00	4.507E-05	8.681E-07	4.026E-06
**0.5**	-2.187E-03	-4.654E-03	0.000E+00	7.129E-05	2.132E-06	6.561E-06
**0.6**	-3.466E-03	-7.673E-03	-1.000E-06	8.490E-05	2.243E-06	8.500E-06
**0.7**	-5.057E-03	-1.165E-02	0.000E+00	7.055E-05	-5.858E-07	9.087E-06
**0.8**	-6.951E-03	-1.666E-02	0.000E+00	8.381E-05	2.499E-06	8.493E-06
**0.9**	-9.128E-03	-2.278E-02	0.000E+00	3.689E-05	2.206E-06	3.544E-05
**1.0**	-1.157E-02	-3.005E-02	0.000E+00	1.393E-04	2.088E-06	2.158E-05

The comparison of absolute errors given in Table 11 above shows that the absolute errors generated by proposed method are quite smaller than errors generated by available numerical methods i.e. VIM and HPM while the generated errors are in quite good comparison with the errors generated by Haar Wavelet method [8]. However, by increasing the number of basis functions, the results can be made very close to the exact results and results obtained by Haar Wavelet method.

### Implementation of proposed scheme using Bernstein Polynomial basis function

**Problem 01.** The same system of nonlinear boundary value problem along with boundary value conditions is considered as for Log Sigmoid basis function which is:

$$x^{\prime \prime }-\delta x^{4}=0$$ (18)

The boundary conditions are:

$$x^{\prime }(0)=0\;\mathrm{and}\;x(1)=1$$

All the settings and parameters remains the same as used for Log Sigmoid basis function for tuning the algorithms. B-Polynomial degree of 8 i.e. *k* = 8 is opted to use.

Two cases for Eq (18) have been treated by considering *δ* = 0.6 and *δ* = 2.0. The fitness functions formulated for Eq (18) for both the cases i.e. for *δ* = 0.6 and *δ* = 2.0 are as follows:

$$\varepsilon_{j1}=\frac{1}{11}\sum_{i=1}^{11}\left(x\prime \prime \left(t_{i}\right)-0.6x^{4}{\left(t_{i}\right)}^{2}\right)+\frac{1}{2}\left({\left(x^{\prime }\left(0\right)\right)}^{2}+{\left(x\left(1\right)-1\right)}^{2}\right)$$ (19)

$$\varepsilon_{j2}=\frac{1}{11}\sum_{i=1}^{11}\left(x\prime \prime \left(t_{i}\right)-2x^{4}{\left(t_{i}\right)}^{2}\right)+\frac{1}{2}\left({\left(x^{\prime }\left(0\right)\right)}^{2}+{\left(x\left(1\right)-1\right)}^{2}\right)$$ (20)

Where *x*(*t*), *x*′(*t*), *x*″(*t*) are same as defined by Eqs (10–12) respectively.

The fitness functions in Eqs (19 and 20) are functions of unknown parameters (*α*_0_, *α*_1_,…..*α*_*k*_) that are necessary to be known for obtaining the optimum numerical solution to given nonlinear ODE. The values of unknown parameters obtained by GA, IPA and GA-IPA are provided in Tables 12 and 13 for case 1 and case 2 respectively. The approximate solutions achieved by GA, IPA and GA-IPA for both the cases are provided in Tables 14 and 15. The comparisons of absolute errors necessary to demonstrate the applicability of the approach are provided in Tables 16 and 17.

**Table 12:** Unknown parameters achieved by G.A, IPA and GA-IPA for δ = 0.6.

Parameters	G.A	IPA	G.A-IPA
α_0_	0.834608	0.834543	0.834543
α_1_	0.834592	0.834543	0.834543
α_2_	0.839776	0.839740	0.839740
α_3_	0.850157	0.850133	0.850133
α_4_	0.866002	0.865971	0.865971
α_5_	0.887716	0.887714	0.887714
α_6_	0.916234	0.916226	0.916226
α_7_	0.952756	0.952756	0.952756
α_8_	0.999994	1.000000	1.000000

**Table 13:** Unknown parameters achieved by G.A, IPA and GA-IPA for δ = 2.0.

Parameters	G.A	IPA	G.A-IPA
α_0_	0.694323	0.694332	0.694332
α_1_	0.694327	0.694332	0.694332
α_2_	0.702633	0.702634	0.702634
α_3_	0.719112	0.719144	0.719144
α_4_	0.745115	0.745089	0.745089
α_5_	0.780648	0.780662	0.780662
α_6_	0.830942	0.830928	0.830928
α_7_	0.897625	0.897607	0.897607
α_8_	1.000025	0.999998	0.999998

**Table 14:** Approximate solutions by numerical methods and proposed method for δ = 0.6.

	Numerical Methods				Proposed Method x(t)		
t	x_Maple_	x_GA_	x_HPM_	x_Haar_	G.A	IPA	G.A-IPA
**0.0**	0.834542	0.963536	0.640000	0.834543	0.834608	0.834543	0.834543
**0.2**	0.840390	0.964009	0.652096	0.840391	0.840434	0.840391	0.840391
**0.4**	0.858269	0.965742	0.689536	0.858269	0.858297	0.858269	0.858269
**0.6**	0.889247	0.969893	0.755776	0.889248	0.889262	0.889248	0.889248
**0.8**	0.935346	0.979233	0.866576	0.935346	0.935349	0.935346	0.935346

**Table 15:** Approximate solutions by numerical methods and proposed method for δ = 2.0.

	Numerical Methods				Proposed Method x(t)		
t	x_Maple_	x_GA_	x_HPM_	x_Haar_	G.A	IPA	G.A-IPA
**0.0**	0.694318	0.968771	0.666667	0.694362	0.694323	0.694332	0.694332
**0.2**	0.703698	0.968804	0.625600	0.703739	0.703700	0.703707	0.703707
**0.4**	0.732894	0.969008	0.489600	0.732927	0.732897	0.732902	0.732902
**0.6**	0.785488	0.970024	0.220267	0.785510	0.785493	0.785490	0.785490
**0.8**	0.869161	0.975059	0.246400	0.869176	0.869179	0.869166	0.869166

**Table 16:** Comparison of absolute errors between numerical methods and proposed method for δ = 0.6.

	Numerical Methods [x_Exact_—x(t)]			Proposed Method [x_Exact_—x(t)]		
t	x_GA_	x_HPM_	x_Haar_	G.A	IPA	G.A-IPA
**0.0**	-1.290E-01	1.945E-01	-1.000E-06	-6.614E-05	-1.035E-06	-1.035E-06
**0.2**	-1.236E-01	1.883E-01	-1.000E-06	-4.390E-05	-8.642E-07	-8.642E-07
**0.4**	-1.075E-01	1.687E-01	0.000E+00	-2.804E-05	-4.940E-07	-4.940E-07
**0.6**	-8.065E-02	1.335E-01	-1.000E-06	-1.483E-05	-6.802E-07	-6.802E-07
**0.8**	-4.389E-02	6.877E-02	0.000E+00	-3.439E-06	-3.669E-07	-3.669E-07

By comparing the absolute errors generated by numerical methods and proposed method, it is established that the proposed method provided much excellent results than GA and HPM methods while results are in sharp agreement with the results provided by Haar Wavelet Technique.

**Table 17:** Comparison of absolute errors between numerical methods and proposed method for δ = 2.0.

	Numerical Methods [x_Exact_—x(t)]			Proposed Method [x_Exact_—x(t)]		
t	x_GA_	x_HPM_	x_Haar_	G.A	IPA	G.A-IPA
**0.0**	-2.745E-01	2.765E-02	-4.400E-05	-5.175E-06	-1.416E-05	-1.416E-05
**0.2**	-2.651E-01	7.810E-02	-4.100E-05	-1.625E-06	-8.509E-06	-8.510E-06
**0.4**	-2.361E-01	2.433E-01	-3.300E-05	-3.321E-06	-7.658E-06	-7.658E-06
**0.6**	-1.845E-01	5.652E-01	-2.200E-05	-4.923E-06	-1.759E-06	-1.759E-06
**0.8**	-1.059E-01	6.228E-01	-1.500E-05	-1.823E-05	-4.856E-06	-4.857E-06

From the comparison of results shown in Table 17, it is clear that the proposed method has provided optimum results as compared to numerical methods GA, HPM and Haar Wavelet method.

**Problem 02.** Again the same system, considered for Log Sigmoid basis function, which is represented by the nonlinear initial value problem of Eq (2) is taken into account as:

$$x^{\prime }+x+\delta x^{4}=0$$ (21)

The initial condition is

x(0)=1

For tuning the algorithms using Bernstein Polynomial basis function, the settings and parameters used are same as for Log Sigmoid basis function shown under section 4.1. The fitness function formulated for Eq (21) is given by:

$$\varepsilon_{j}=\frac{1}{11}\sum_{i=1}^{11}\left({\left(x^{\prime }\left(t_{i}\right)+x\left(t_{i}\right)+\delta x^{4}\left(t_{i}\right)\right)}^{2}\right)+\left({\left(x\left(0\right)-1\right)}^{2}\right)$$ (22)

Where *x*(*t*) and *x*′(*t*) are same as defined by Eqs (10 and 11) respectively.

The values of unknown parameters corresponding to minimum *ε*_*j*_ for different values of δ are omitted in this case. The results provided by GA, IPA and GA-IPA are shown in Table 18. The comparison of absolute errors generated by proposed method is made with available numerical methods and is provided in Table 19.

**Table 18:** Approximate solutions by numerical methods and proposed method for different and t = 0.5.

Numerical Methods					Proposed Method x(t)		
δ	x_Exact_	x_VIM_	x_HPM_	x_Haar_	G.A	IPA	GA-IPA
**0.0**	0.606531	0.606531	0.606531	0.606531	0.606532	0.606531	0.606531
**0.1**	0.591591	0.591617	0.591638	0.591592	0.591594	0.591591	0.591591
**0.2**	0.578023	0.578207	0.578371	0.578023	0.578030	0.578022	0.578022
**0.3**	0.565620	0.566185	0.566732	0.565620	0.565616	0.565616	0.565616
**0.4**	0.554217	0.555440	0.556720	0.554217	0.554210	0.554214	0.554209
**0.5**	0.543681	0.545868	0.548335	0.543681	0.543667	0.543670	0.543667
**0.6**	0.533903	0.537369	0.541576	0.533904	0.533882	0.533890	0.533882
**0.7**	0.524793	0.529850	0.536445	0.524793	0.524761	0.524761	0.524761
**0.8**	0.516275	0.523226	0.532940	0.516275	0.516231	0.516230	0.516230
**0.9**	0.508284	0.517412	0.531062	0.508284	0.508224	0.508231	0.508224
**1.0**	0.500765	0.512333	0.530812	0.500765	0.500690	0.500689	0.500689

**Table 19:** Comparison of absolute errors for different δ and t = 0.5.

	Numerical Methods [x_Exact_—x(t)]			Proposed Method[x_Exact_—x(t)]		
δ	x_VIM_	x_HPM_	x_Haar_	G.A	IPA	G.A-IPA
**0**	0.000E+00	0.000E+00	0.000E+00	-9.393E-07	3.403E-07	3.403E-07
**0.1**	-2.600E-05	-4.700E-05	-1.000E-06	-3.308E-06	-8.758E-08	-8.758E-08
**0.2**	-1.840E-04	-3.480E-04	0.000E+00	-6.922E-06	1.402E-06	1.402E-06
**0.3**	-5.650E-04	-1.112E-03	0.000E+00	3.778E-06	4.242E-06	4.242E-06
**0.4**	-1.223E-03	-2.503E-03	0.000E+00	7.376E-06	3.236E-06	8.236E-06
**0.5**	-2.187E-03	-4.654E-03	0.000E+00	1.383E-05	1.145E-05	1.383E-05
**0.6**	-3.466E-03	-7.673E-03	-1.000E-06	2.144E-05	1.321E-05	2.144E-05
**0.7**	-5.057E-03	-1.165E-02	0.000E+00	3.188E-05	3.183E-05	3.183E-05
**0.8**	-6.951E-03	-1.666E-02	0.000E+00	4.400E-05	4.479E-05	4.479E-05
**0.9**	-9.128E-03	-2.278E-02	0.000E+00	5.967E-05	5.326E-05	5.967E-05
**1.0**	-1.157E-02	-3.005E-02	0.000E+00	7.487E-05	7.605E-05	7.605E-05

It is evident from the comparison of absolute errors given above in Table 19 that the absolute errors generated by proposed method are quite smaller than errors generated by available numerical methods i.e. VIM and HPM which testifies the viability and affirms the accuracy of proposed approach while the generated absolute errors are in good comparison with the errors generated by Haar Wavelet method [8] which can be further improved by increasing the degree *k* of basis function.

## Statistical analysis for proposed approach

In this section, statistical analysis has been performed for nonlinear heat transfer problems considered in this paper both with log sigmoid and B-Polynomial approaches to demonstrate the stability and authenticity of the proposed methodology. For this sake, 10 independent runs of the proposed methodology are performed without any deviation in the parametric settings of the problem as shown in Tables 2 and 9 of this paper. For statistical analysis, the parameters considered are the best values of the absolute error, the worst values of the absolute error, mean of the absolute error and the standard deviation (STD) of the absolute error for all the schemes i.e. GA, IPA and GA-IPA. The best and worst values of the absolute errors are replicate to the minimum and maximum errors respectively while Mean and standard deviation (STD) of the absolute errors determines the degree of variation in the final results which ultimately replicate the stability of the presented approach. The statistical analysis for example 01 is carried out for both the case i.e for *δ* = 0.6 and *δ* = 2.0 while for example 02 to avoid increase in the length of the paper, the statistical analysis is carried out only for *δ* = 0.5 and *δ* = 1.0 both for log sigmoid and B-Polynomial approaches.

**Fig 1:**
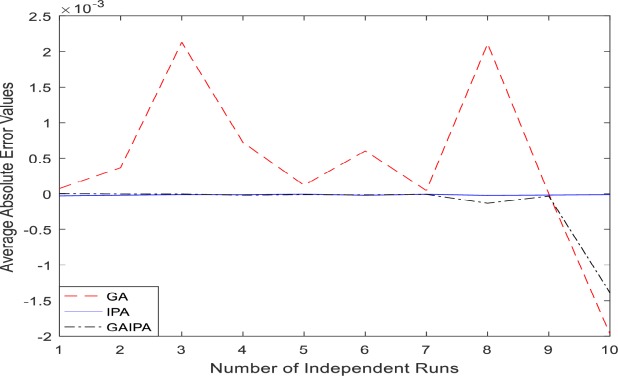
Graphical representation of average absolute errors for Example #01 when δ = 0.6 using Log Sigmoid.

**Fig 2:**
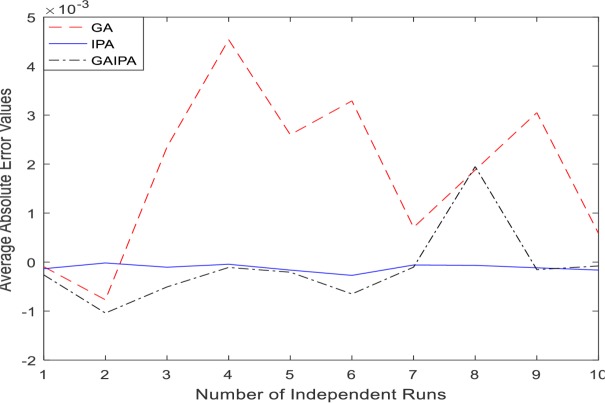
Graphical representation of average absolute errors for Example #01 when δ = 2.0 using Log Sigmoid.

**Fig 3:**
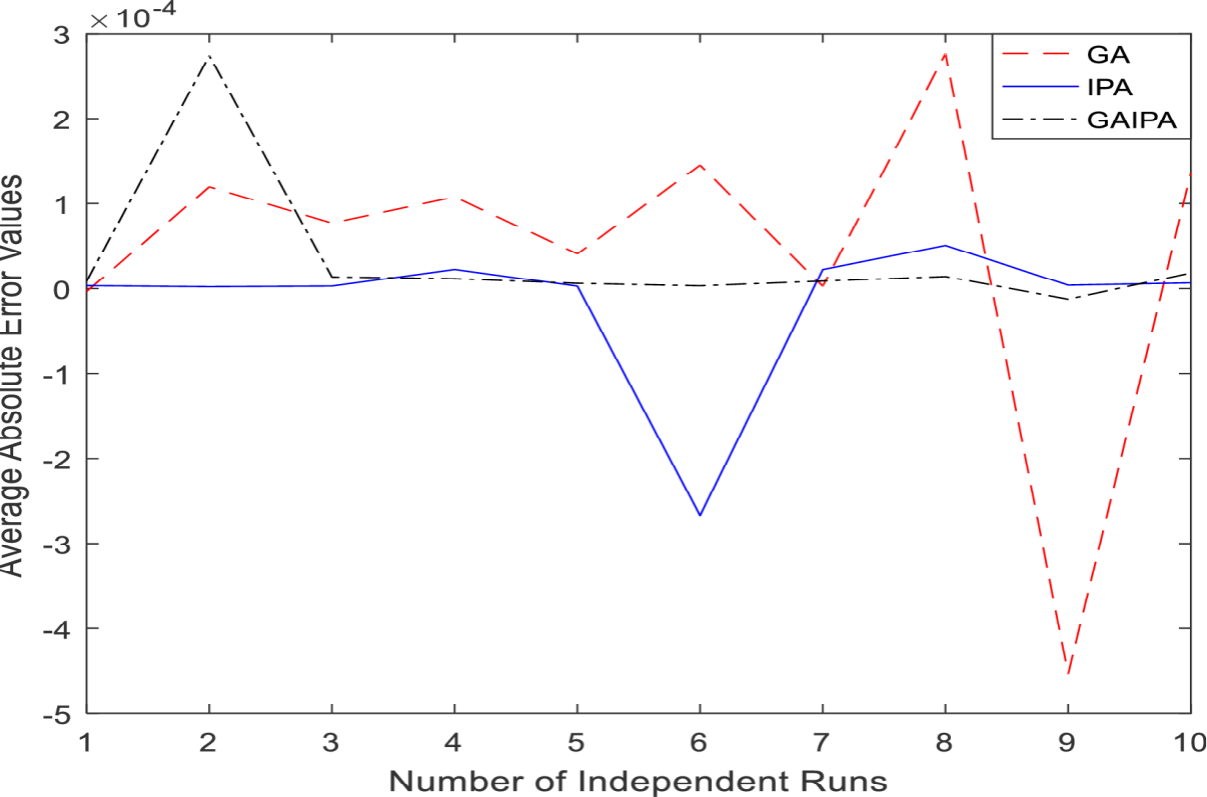
Graphical representation of average absolute errors for Example #02 when δ = 0.5 using Log Sigmoid.

**Fig 4:**
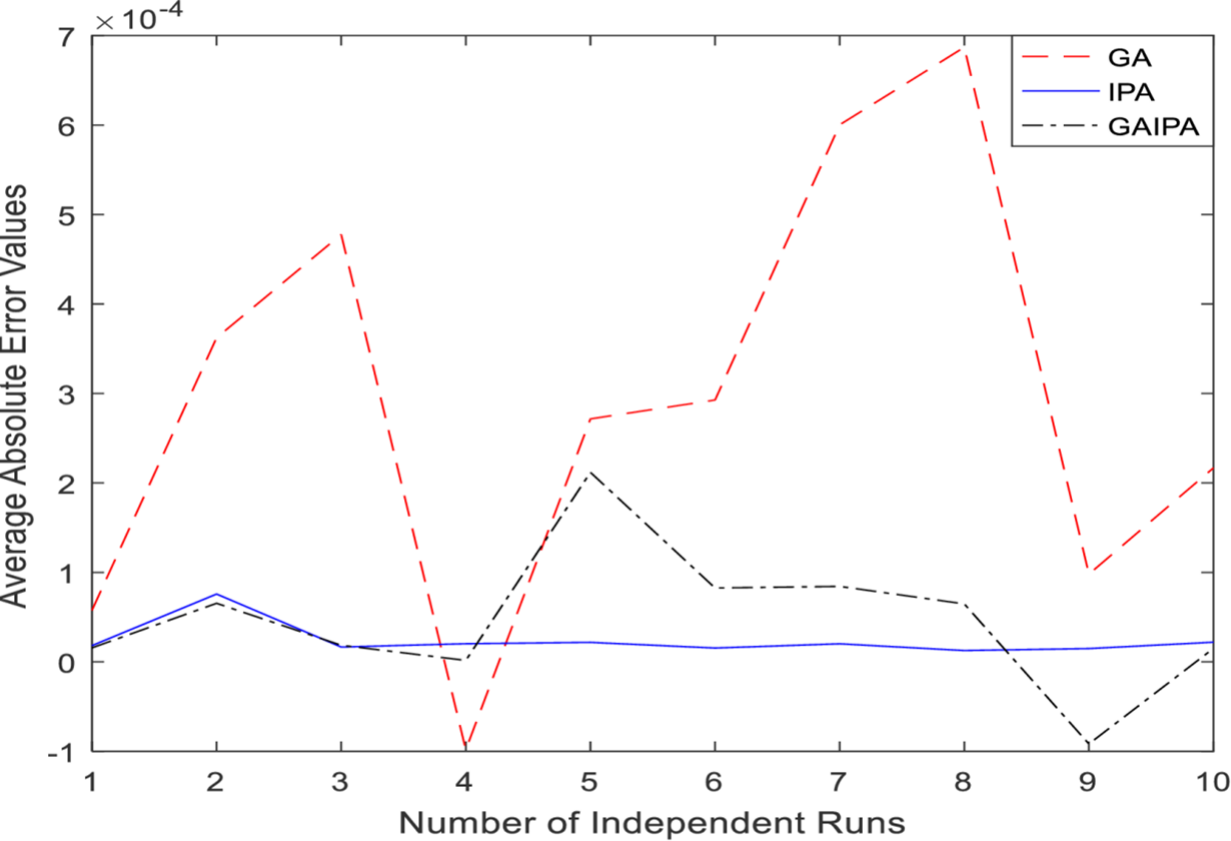
Graphical representation of average absolute errors for Example #02 when δ = 1.0 using Log Sigmoid.

**Fig 5:**
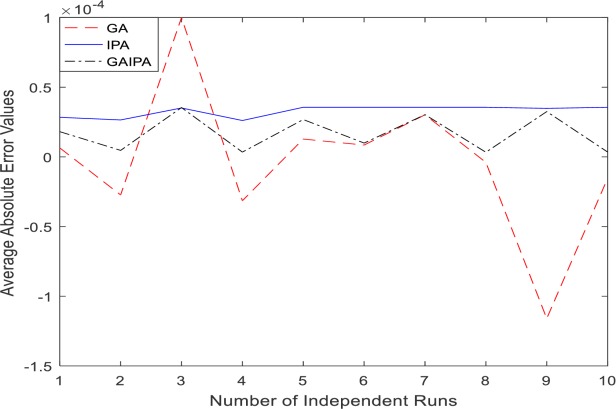
Graphical representation of average absolute errors for Example #01 when δ = 0.6 using B-Polynomial.

**Fig 6:**
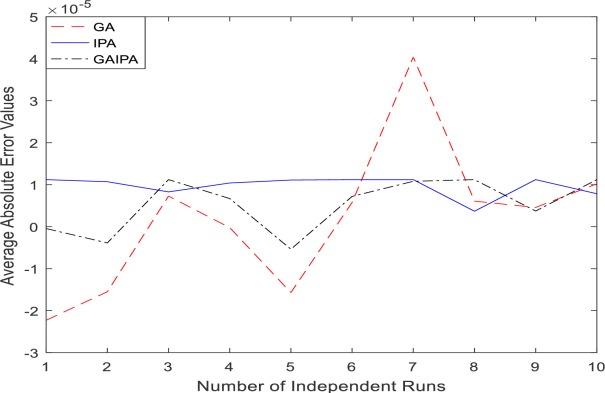
Graphical representation of average absolute errors for Example #01 when δ = 2.0 using B-Polynomial.

**Fig 7:**
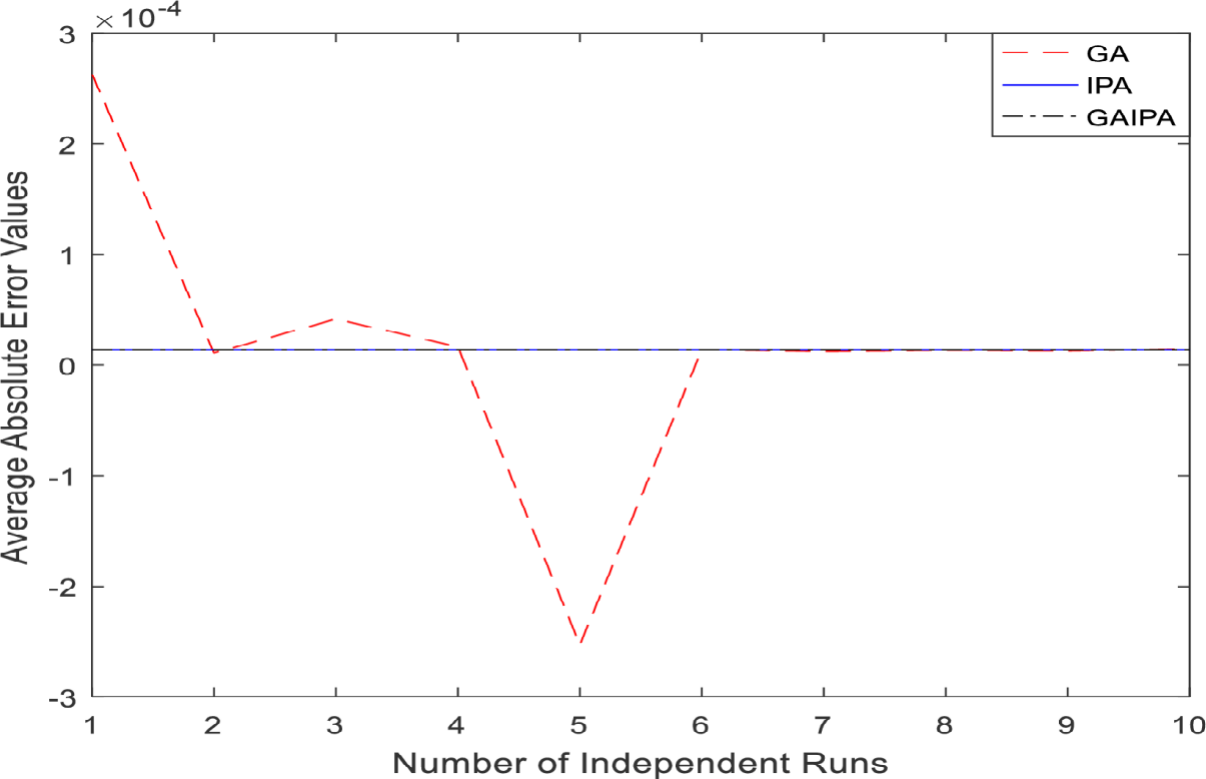
Graphical representation of average absolute errors for Example #02 when δ = 0.5 using B-Polynomial.

**Fig 8:**
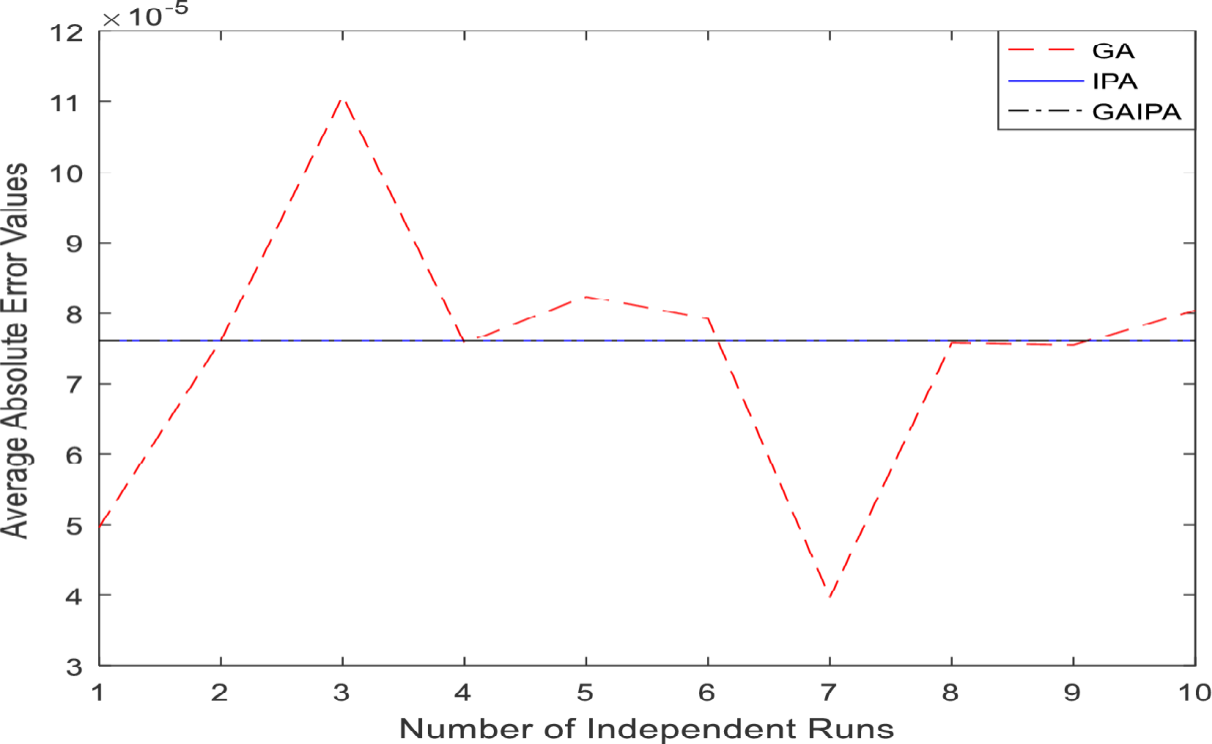
Graphical representation of average absolute errors for Example #02 when δ = 1.0 using B-Polynomial.

The graphical representation for the numerical results of average absolute errors for 10 independent runs are shown in Figs 1–8 both for log sigmoid and B-Polynomials based approaches.The results of the statistical analysis in terms of best values, the worst values, mean and the standard deviation (STD) of the average absolute errors for GA, IPA and GA-IPA schemes for heat transfer problems are presented in Table 20.

**Table 20:** Statistical analysis for heat transfer problems.

	Scheme	Hybridization with Log Sigmoid (k = 10)				Hybridization with B-Polynomial (k = 08)			
		Best	Worst	Mean	STD	Best	Worst	Mean	STD
**Example 01** **(Case-I)** **(δ = 0.6)**	**GA**	-1.97E-03	2.13E-03	4.21E-04	1.16E-03	-1.16E-04	1.00E-04	-3.62E-06	5.43E-05
	**IPA**	-2.89E-05	-5.38E-06	-1.55E-05	7.30E-06	2.61Ez-05	3.56E-05	3.29E-05	4.11E-06
	**GA-IPA**	-1.39E-03	3.02E-06	-1.61E-04	4.34E-04	3.34E-06	3.54E-05	1.68E-05	1.34E-05
**Example 01** **(Case-II)** **(δ = 2.0)**	**GA**	-7.69E-04	4.54E-03	1.81E-03	1.67E-03	-2.23E-05	4.04E-05	2.05E-06	1.77E-05
	**IPA**	-2.71E-04	-1.75E-05	-1.14E-04	7.42E-05	3.67E-06	1.12E-05	9.68E-06	2.46E-06
	**GA-IPA**	-1.04E-03	1.95E-03	-1.16E-04	7.88E-04	-5.37E-06	1.12E-05	5.23E-06	6.44E-06
**Example 02** **(Case-I)** **(δ = 0.5)**	**GA**	-4.53E-04	2.77E-04	4.49E-05	1.93E-04	-2.53E-04	2.63E-04	1.46E-05	1.22E-04
	**IPA**	-2.67E-04	5.08E-05	-1.49E-05	8.98E-05	1.38E-05	1.38E-05	1.38E-05	3.67E-12
	**GA-IPA**	-1.24E-05	2.74E-04	3.46E-05	8.45E-05	1.38E-05	1.38E-05	1.38E-05	4.84E-12
**Example 02** **(Case-II)** **(δ = 1.0)**	**GA**	-9.82E-05	6.87E-04	2.97E-04	2.45E-04	3.97E-05	1.11E-04	7.45E-05	1.91E-05
	**IPA**	1.26E-05	7.58E-05	2.38E-05	1.85E-05	7.61E-05	7.61E-05	7.61E-05	1.97E-11
	**GA-IPA**	-9.17E-05	2.12E-04	4.68E-05	7.79E-05	7.61E-05	7.61E-05	7.61E-05	5.10E-12

From Table 20, It can be seen that the mean of the average absolute error lies within the range 10^−05^ to 10^−03^ while the STD of the absolute error lies within the range 10^−06^ to 10^−03^ for Log Sigmoid based approach. Similarly, the mean of the average absolute error lies within the range 10^−06^ to 10^−05^ while the STD of the absolute error lies within the range 10^−12^ to 10^−04^ for B-Polynomial based approach. The very close ranges of mean and STD determines the close deviation from the results which is a measure of the stability and authenticity of the proposed approach.

## Conclusion

On the basis of simulations and results provided by the proposed approach, it is observed that the proposed hybrid heuristic approach of GA-IPA out performs the other existing solutions for the nonlinear heat transfer equations. Further, the proposed scheme shows the supremacy over numerical methods i.e. Generalized Approximation, Homotopy Perturbation Method and Variational Iteration Method. In some cases the results are even more satisfactory than results provided by the numerical method Haar Wavelet Quasilinearization Technique. In this connection, it is established that the proposed approach is a good and trusty alternative approach for researchers for solving nonlinear problems in engineering and applied sciences domain.
